# Illnesses and hardship financing in India: an evaluation of inpatient and outpatient cases, 2014-18

**DOI:** 10.1186/s12889-023-15062-7

**Published:** 2023-01-30

**Authors:** Arya Rachel Thomas, Umakant Dash, Santosh Kumar Sahu

**Affiliations:** 1 Department of Humanities and Social Sciences, Indian Institute of Technology Madras, Chennai, India; 2 Institute of Rural Management Anand (IRMA), Anand, India; 3 Department of Humanities and Social Sciences, Indian Institute of Technology Madras, Chennai, India

**Keywords:** Hardship financing, Inpatient care, Outpatient care, Government intervention

## Abstract

**Background:**

Progress towards universal health coverage requires strengthening the country's health system. In developing countries, the increasing disease burden puts a lot of stress on scarce household finances. However, this burden is not the same for everyone. The economic burden varies across the disease groups and care levels. Government intervention is vital in formulating policies in addressing financial distress at the household level. In India, even when outpatient care forms a significant proportion of out-of-pocket expenditure, government schemes focus on reducing household expenditure on inpatient care alone. Thus, people resort to hardship financing practices like informal borrowing or selling of assets in the event of health shocks. In this context, the present study aims to identify the disease(s) that correlates with maximum hardship financing for outpatients and inpatients and to understand the change in hardship financing over time.

**Methods:**

We used two waves of National Sample Survey Organisation’s data on social consumption on health- the 71^st^ and the 75^th^ rounds. Descriptive statistics are reported, and logistic regression is carried out to explain the adjusted impact of illness on hardship financing. Pooled logistic regression of the two rounds is estimated for inpatients and outpatients. Marginal effects are reported to study the changes in hardship financing over time.

**Results:**

The results suggest that cancer had the maximum likelihood of causing hardship financing in India for both inpatients (Odds ratio 2.41; 95% Confidence Interval (CI): 2.03 - 2.86 (71^st^ round), 2.54; 95% CI: 2.21 - 2.93 (75^th^ round)) and outpatients (Odds ratio 6.11; 95% CI: 2.95 - 12.64 (71^st^ round), 3.07; 95% CI: 2.14 - 4.40 (75^th^ round)). In 2018, for outpatients, the hardship financing for health care needs was higher at public health facilities, compared to private health facilities (Odds ratio 0.72; 95% CI: 0.62 - 0.83 (75^th^ round). The marginal effects model of pooled cross-section analysis reveals that from 2014 to 2018, the hardship financing had decreased for inpatients (Odds ratio 0.747; 95% CI:0.80 - -0.70), whereas it had increased for outpatients (Odds ratio 0.0126; 95% CI: 0.01 - 0.02). Our results also show that the likelihood of resorting to hardship financing for illness among women was lesser than that of men.

**Conclusion:**

Government intervention is quintessential to decrease the hardship financing caused by cancer. The intra-household inequalities play an important role in explaining their hardship financing strategies. We suggest the need for more financial risk protection for outpatient care to address hardship financing.

**Supplementary Information:**

The online version contains supplementary material available at 10.1186/s12889-023-15062-7.

## Introduction

In India, National Health Accounts for 2018-19 shows that the total health expenditure (THE) is estimated to be 3.16% of GDP (Rs.5,96,440 crores). Out of which, the government health expenditure accounts for 40.61% of THE. At the same time, about 48.21% of THE is met out of pocket by the households. Out-of-pocket expenditure (OOPE) also accounts for 53.32% of current health expenditure [1]. The OOPE is catastrophic when the household has to decrease its basic expenses to meet the cost of health care [2, 3]. At an arbitrarily set threshold of 10%, the incidence of household catastrophic health expenditure for households who accessed private hospitals are 43.99% [4]. Around one-third of all households suffering from non-communicable diseases (NCDs) have spent beyond the 10% threshold [5].

Globally, the rapid demographic transition from high to low birth and death rates is followed by a parallel epidemiological change, and the burden of diseases shifts from infectious to NCDs [6]. However, in some low and middle-income countries (LMIC), like India, there exists a dual burden of diseases [7–9], i.e. NCDs and the continuing prevalence of communicable diseases [10] that seem to co-exist. LMIC's share of the global burden of diseases (GBD) is disproportionately higher than the rich countries. However, their health spending is much lesser. About 90% of the GBD befalls developing countries, which will take a toll on scarce household and government resources [11].

A crucial attempt to bring financial protection to the poor was the introduction of the Rashtriya Swasthya Bhima Yojana (RSBY) in 2008 [12]. Along with many other drawbacks like low enrolment and utilisation, the scheme couldn't reduce people's OOPE. Like other schemes in the past, RSBY also focused on decreasing OOPE for inpatient care alone when OOPE is primarily borne by spending on drugs and outpatient care [13–15]. Set out to straighten the drawbacks of RSBY, in 2018, Ayushman Bharat was launched under the recommendation of the National Health Policy, 2017. The two components of this scheme are Pradhan Mantri Jan Arogya Yojana (PMJAY) and Health and Wellness Centre (HWC). PMJAY focuses on providing insurance for secondary and tertiary hospitalisation cases [16]. The financial coverage PMJAY aims to provide is 17 times better than RSBY [17]. The HWC aims to strengthen primary healthcare, including free preventive and basic curative services, primarily focusing on immunisation, maternal and child health, and communicable diseases [18]. The expanded range of services offered by HWCs is given in the appendix ( see Additional file 1: Appendix Table 1) [19].

**Table 1:** Descriptive statistics of outpatient cases

	**71**^**st**^ **Round** **2014(A)**		**75**^**th**^ **round** **2018 (B)**		**Pooled(A+B)**	
	**N**	**%**	**N**	**%**	**N**	**%**
**Ailment**						
Infection	6,547	26.57	9,144	30.59	15,691	24.15
Cancer	67	0.22	355	0.35	422	0.65
Blood Disease	201	0.82	363	0.88	564	0.87
Endocrine	3,945	13.62	7,389	16.43	11,334	17.44
Psychiatric	1,229	4.27	1,742	3.89	2,971	4.57
Genito-urinary	427	1.70	599	1.22	1,026	1.58
Eye	326	1.33	385	0.97	711	1.09
Ear	148	0.61	148	0.34	296	0.46
Cardio-vascular	4,463	14.55	8,060	18.5	12,523	19.27
Respiratory	3,644	12.86	3,666	9.45	7,310	11.25
Gastro-intestinal	1,475	6.27	1,539	4.36	3,014	4.64
Skin	600	2.53	702	2.22	1,302	2.00
Musculo-Skeletal	2,650	10.79	2,867	8.13	5,517	8.49
Obstetric	95	0.26	152	0.27	247	0.38
Injuries	358	1.28	573	1.12	931	1.43
Others	588	2.33	533	1.28	1,121	1.73
**Gender**						
Male	12,068	44.72	17,946	45.81	30,014	46.19
Female	14,695	55.28	20,271	54.19	34,966	53.81
**Education Status**						
Illiterate	10,025	37.83	13,033	34.48	23,058	35.49
Less than primary education	3,690	14.42	5,285	15.37	8,975	13.81
Completed primary education	3,392	13.81	4,762	12.93	8,154	12.55
Middle school completed	3,336	12.29	4,631	11.8	7,967	12.26
Completed secondary or higher secondary	4,425	15.57	7,177	17.91	11,602	17.86
Graduation and above	1,894	6.09	3,325	7.50	5,219	8.03
**Type of Employment of Household**						
Casual labour	3,549	45.66	7,206	23.27	10,755	16.55
Self-employed	12,093	45.66	17,332	45.43	29,425	45.28
Regular wage	6,931	21.78	9,728	20.54	16,659	25.64
Household without income	4,190	18.43	3,951	10.76	8,141	12.53
**Income Quintile**						
Poorest	2,939	17.13	4,555	19.48	7,494	11.53
Poor	4,728	22.88	4,507	15.63	9,235	14.21
Middle	3,285	14.24	7,230	22.01	10,515	16.18
Rich	6,053	20.26	7,591	17.81	13,644	21.00
Richest	9,758	25.49	14,334	25.06	24,092	37.08
**Living Condition Index**						
Low	11,598	50.48	21,226	63.14	32,824	50.51
High	15,165	49.52	16,991	36.86	32,156	49.00
**Chronic Diseases**						
Suffered	15,315	55.55	24,139	55.22	39,454	60.72
Did not suffer	11,448	44.45	14,074	44.78	25,522	39.28
**Location**						
Urban	13808	39.17	18,871	37.90	32,679	50.29
Rural	12955	60.83	19,346	62.10	32,301	49.71
**Religion**						
Hinduism	20,199	78.58	27,780	77.19	47,979	73.84
Islam	4,032	13.45	6,605	15.41	10,637	16.37
Christianity	1,433	4.52	2,171	3.90	3,604	5.55
Others	1,099	3.45	1,661	3.50	2,760	4.25
**Social Group**						
SC/ST	6,033	22.18	8,220	22.74	14,253	21.93
OBC	11,378	44.82	15,419	42.33	26,797	41.24
Others	9,352	32.99	14,578	34.92	23,930	36.83
**Type of Medical Institution**						
Public	7,024	25.08	12,465	30.01	19,489	29.99
Private	19,739	74.92	25,750	69.99	45,489	70.01
**Age Group**						
Children	5,584	19.50	6,784	18.5	12,368	19.03
Working age	13,714	53.49	18,783	52.69	32,497	50.01
Elderly	7,465	27.01	12,646	28.81	20,111	30.95
**State Regions**						
North zone	4,820	18.07	7,499	21.71	12,319	18.96
South zone	9,933	39.88	12,339	30.82	22,272	34.28
East zone	4,844	20.76	6,954	21.54	11,798	18.16
West zone	3,952	14.65	6,372	18.56	10,324	15.89
Central zone	1,219	4.16	1,814	4.33	3,033	4.67
North-east zone	756	1.04	1,083	0.92	1,839	2.83
Union Territory	1,239	1.45	2,156	2.11	3,395	5.22
**Free Advice**						
Yes	6,549	23.50	12,277	30.42	18,826	28.98
No	20,209	76.50	25,917	69.58	46,126	71.02
**Marital Status**						
Unmarried	11,272	42.27	15,223	42.77	26,495	40.78
Married	15,491	57.73	22,990	57.23	38,481	59.22

Source: Based on the authors’ computation from NSSO 71^st^ and 75^th^ round unit level data. The 'N' represents the unweighted sample size of each category of the independent variable. '%' is the weighted percentage of each category of the independent variable for the cross-sectional data sets and the unweighted percentage for the pooled data set.

Nevertheless, insuring expenditure against hospitalisation continues to be over-emphasised in India, and the PMJAY component has received priority over the HWC [20]. Also, the financing of health by the government is low in the country [21]. Thus, the overall financial protection offered by the government schemes is limited, putting a lot of stress on the limited household finances.

### Evidence against the theory of full insurance

The coping strategies adopted by the households to finance health can be ex-ante or ex-post [22, 23]. Ex-ante strategies are risk management mechanisms to insure households against unanticipated events like illness shocks and thereby control income variability [24]. As per the theory of full insurance, a household is perfectly insured if, among other factors, the idiosyncratic unanticipated health shocks have no impact on household consumption [25, 26].

Some studies validate the existence of full insurance and the capacity of low-income households to insure consumption against health shocks decently well [22, 27, 28].

However, some studies find evidence against full insurance, especially when households face severe health shocks. Cochrane [26] finds evidence that in the United States, where disease episodes lasting more than 100 days and involuntary unemployment will hurt consumption levels. Gertler et al. [29] find that a decline in the health of the adult from significant health shocks results in their consumption decline.

In developing countries, financial constraints, coupled with the unpredictability of illness episodes, only a minority of the population resort to the ex-ante methods of insuring to meet the health shocks. The poor people who struggle to meet daily subsistence food consumption are incapable of insuring themselves from unexpected illnesses. In 2018, only about 14.1% of the rural and 19.1 % of the urban populations had insurance coverage [30]. Therefore people resort to ex-post coping mechanisms like using income savings, selling livestock or assets, and borrowing from family, friends or money lenders to smoothen consumption expenditure [24].

### Measurement of health-related economic hardship

There are two approaches to examining health-related economic hardship, catastrophic health expenditure and hardship financing [31, 32]. When the OOPE on the household's health is above and beyond an arbitrarily set threshold of 10% or 40%, the household suffers from catastrophic health expenditure [5, 32–35].

An alternate approach is to assess the extent of hardship financing resulting from health shock. The various direct and indirect costs of seeking treatment force people to dissave, borrow, sell assets or undergo other informal mechanisms to meet the expenses [36, 37]. The inefficient government interventions force people to resort to these informal coping strategies [38]. Evidence suggests that about one-quarter of households in 40 LMIC resort to hardship financing [31]. Dercon finds that the risk protection offered by informal mechanisms is limited and exposes poor people to severe financial shocks [39]. Flores shows that in India, about three-quarters of healthcare costs in rural regions and two-thirds in urban areas are borne by income savings, borrowing, selling of assets and transfers [40]. Evidence suggests that informal transactions and mutual insurance primarily occur among family and friends [23, 38, 41–43]. Some studies identify borrowing as the most common strategy adopted to face healthcare shocks [31, 37, 44], and Islam and Maitra [45] identify selling livestock as essential. However, these strategies of borrowing and selling vary across the gender of the ill person. Across all age groups in Indian households, resorting to hardship financing for women's health needs is much lower than men's [46, 47].

### Evidence of hardship financing in India

In India, many works have studied the impact of illness on hardship financing. Kastor and Mohanty assess the effects of diseases on hardship financing [32]. However, the study is cross-sectional and limited to inpatient care. Binnendijik et al. studied how various illnesses impact hardship financing for outpatient and inpatient care, but the investigation is limited to the poor in rural Orissa [48]. John and Kumar did a similar survey of the rural poor in Chhattisgarh [49]. Yadav et al., in 2019, used NSSO 71st round data to examine tuberculosis's impact on hardship financing [50]. Similarly, in 2021, Yadav et al. explored the effect of a single disease, delivery care, in hardship financing using NFHS-4 [51].

In this context, we strive to gather a better understanding of illness-related hardship financing in India. We focus on the impact of unexpected health expenditures, as they could be more difficult for households to insure against [25]. In our study, we define hardship financing as when ex-post informal coping strategies like selling livestock, or assets, informal borrowings from friends and family, and informal money lenders are used to meet the unanticipated financial cost of health shocks [31, 48–51]. The study has two primary objectives. Firstly, the study explores how hardship financing varies across diseases. We aim to identify the disease(s) that cause the largest hardship financing in India for hospitalisation and outpatient cases over time. Secondly, we focus on understanding how hardship financing in the country changed from 2014 to 2018. To our knowledge, it is a first-ever attempt to inquire into the variation in illness-related hardship financing with time in India.

### Data and methods

#### Data source

The study uses large-scale unit level, nationally representative data published by the National Sample Survey Office (NSSO), Ministry of Statistics & Programme Implementation, Government of India. Two rounds of the repeated cross-section National Sample Survey (NSS), namely Survey on Social Consumption (71st round (Jan-Jun2014)) and Social Consumption in India: Health (75th round (July 2017- June 2018)), are used. The data provides quantitative details of the health sector. NSSO uses a stratified multi-stage method of sampling for data collection. Further details of the survey design and data collection can be found in the reports [30, 52].

The different spells of ailment or hospitalisation of the same individual are considered different units in NSSO data. For our analysis, cases of illnesses were categorised based on the level of care; inpatient or outpatient. The individuals who reported hospitalisation 365 days before the survey represented the inpatient cases. The 2014 and 2018 rounds provide information on 57,456 and 93,925 hospitalisation cases, respectively. For outpatients, NSSO has information on individuals who faced ailments (both hospitalisation and non-hospitalisation) in the 15 days before the survey. They provide information on 37,282 ailment cases in 2014 and 43,240 in 2018. This outpatient data is filtered to obtain information on non-hospitalised outpatient cases alone. Since the study focuses on unanticipated health shocks, financial hardship due to health expenses for childbirth is excluded from inpatient and outpatient cases, as they are anticipated expenditures. Thus we conduct our cross-sectional analysis using 40,456 and 63,785 hospitalisation cases and 26,763 and 38,217 outpatient cases for 2014 and 2018, respectively.

### Variables description

**Fig. 1:**
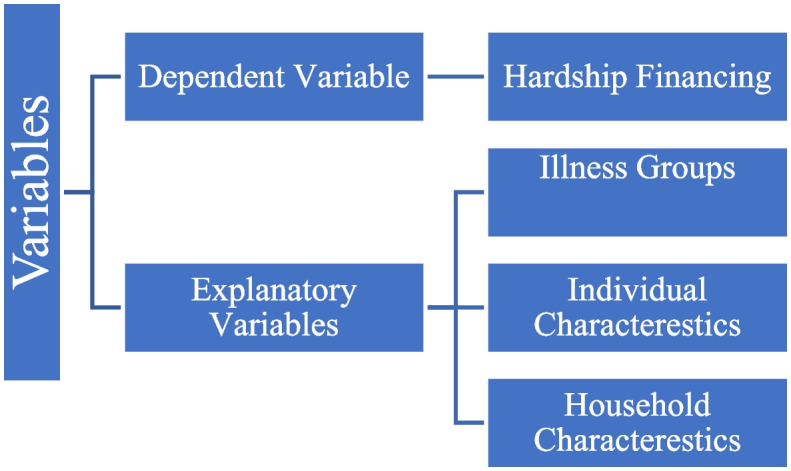
Choice of indicators and variables

Figure 1 gives an outline of the variables used in the study. The dependent variable of the study is hardship financing. NSSO provides information on the sources of financing expenditure for each case of an ailment [30]. They provide information on whether an individual resorted to savings, borrowings, sale of assets, contributions from friends or relatives, or other sources for each case of ailment for inpatient and outpatient care. Income-savings rates are low in LMIC [25, 31] and are considered less burdensome [48]. Therefore, the study does not consider resorting to savings as hardship financing. Hardship financing is a consequence of health shock when an individual has to either borrow money or sell physical assets or contributions from friends and relatives or other sources to meet healthcare expenditures. The study's dependent variable is binary and coded as an individual without hardship financing for the case of illness $(0=no)$ and an individual who resorted to hardship financing for the case of illness $(1=yes).$

Previous studies have identified several factors influencing hardship financing in India [32, 48–51]. The primary explanatory variable of our analyses is the illnesses. We use self-reported disease occurrence as the indicator of individual inpatient and outpatient cases in the study. The diseases (excluding childbirth) were categorised into 16 groups per the nature of ailments reported in NSSO 71^st^ and 75^th^ rounds [30, 52]. The disease categories are infections, cancer, blood-related, Endocrine/Metabolic/Nutritional, Psychiatric and Neurological, Genito-urinary, Eye, Ear, Cardio-vascular, Respiratory, Gastro-intestinal, Skin, Musculoskeletal, Injuries and others. A detailed description of the diseases and their main symptoms as per NSSO reports are provided in the appendix (see Additional file 1: Appendix Table 2).

**Table 2:** Descriptive statistics of inpatient cases

	**71**^**st**^ **round** **2014(A)**		**75**^**th**^ **round** **2018(B)**		**Pooled (A+B)**	
	**N**	**%**	**N**	**%**	**N**	**%**
**Ailment**						
Infection	10,766	25.71	20,362	32.12	31,128	29.86
Cancer	774	1.59	1,243	2.06	2,017	1.93
Blood Disease	825	1.91	1,230	2.22	2,055	1.97
Endocrine	1,149	2.57	1,762	2.58	2,911	2.79
Psychiatric	2,426	5.82	3,550	5.48	5,976	5.73
Genito-urinary	2,711	6.75	3,843	5.94	6,554	6.29
Eye	1,590	4.83	2,091	3.70	3,681	3.53
Ear	183	0.48	336	0.47	519	0.50
Cardio-vascular	3,269	8.46	5,654	8.72	8,923	8.56
Respiratory	2,025	5.01	2,514	3.98	4,539	4.35
Gastro-intestinal	4,739	11.19	6,765	10.03	11,504	11.04
Skin	390	0.94	628	0.93	1,018	0.98
Musculo-Skeletal	1,954	4.98	2,898	4.53	4,852	4.65
Obstetric	1,937	5.58	1,778	3.55	3,715	3.56
Injuries	4,519	11.25	7,697	11.43	12,216	11.72
Others	1,199	2.95	1,434	2.27	2,633	2.53
**Gender**						
Male	20,496	49.41	33,105	51.11	53,601	51.42
Female	19,960	50.59	30,680	48.89	50,640	48.58
**Education Status**						
Illiterate	14,102	37.49	18,864	32.25	32,966	31.62
Less than primary education	5,417	13.33	8,532	13.29	13,949	13.38
Completed primary education	5,138	12.50	8,180	13.82	13,318	12.78
Middle school completed	5,450	13.82	8,593	13.20	14,043	13.47
Completed secondary or higher secondary	7,395	16.85	14,052	20.53	21,447	20.57
Graduation and above	2,954	6.01	5,564	6.91	8,518	8.17
**Type of Employment**						
Casual labour	5,519	15.36	13,044	24.56	18,563	17.81
Self-employed	19,414	48.28	30,533	47.33	49,947	47.91
Regular wage	9,734	20.50	15,157	20.70	24,891	23.88
Household without income	5,789	15.86	5,051	7.40	10,840	10.40
**Income Quintile**						
Poorest	6,067	15.97	10,379	18.11	16,446	15.78
Poor	8,703	21.12	9,856	16.89	18,559	17.80
Middle	5,190	13.31	14,030	22.48	19,220	18.44
Rich	9,064	22.60	11,550	17.53	20,614	19.78
Richest	11,427	27.00	17,970	24.99	29,397	28.20
**Living Condition Index**						
Low	20,094	55.80	36,249	64.00	56,343	54.05
High	20,362	44.20	27,535	36.00	47,897	45.95
Insurance						
Insured	7,951	21.93	14,324	23.13	22,275	21.37
Uninsured	32,505	78.07	49,461	76.87	81,966	78.63
**Chronic Diseases**						
Suffered	8,782	24.83	10,358	19.37	19,140	18.36
Did not suffer	31,674	75.17	53,427	80.63	85,101	81.64
**Location**						
Urban	18,697	64.92	28,266	35.21	46,963	45.00
Rural	21,759	35.08	35,519	64.79	57,278	54.95
**Religion**						
Hinduism	31,208	79.74	48,274	79.23	79,482	76.25
Islam	5,580	13.63	8,695	14.40	14,275	13.69
Christianity	2,268	3.82	4,165	3.58	6,433	6.17
Others	1,400	2.82	2,651	2.79	4,051	4.00
**Social Group**						
SC/ST	11,163	25.15	17,853	25.02	29,016	27.84
OBC	16,181	44.86	25,721	43.8	41,902	40.20
Others	13,112	30.00	20,211	31.19	33,323	31.97
**Type of Medical Institution**						
Public	17,343	38.02	29,346	41.72	46,689	44.79
Private	23,113	61.98	34,439	58.28	57,552	55.21
**Age Group**						
Children	7,120	16.51	11,013	16.60	18,133	17.40
Working age	24,941	61.74	40,130	63.32	65,071	62.42
Elderly	8,395	21.75	12,642	20.08	21,037	20.18
**State Regions**						
North zone	7,428	17.11	11,811	18.94	19,239	18.46
South zone	9,516	34.82	14,992	30.38	24,508	23.51
East zone	7,026	18.22	10,688	20.06	17,714	16.99
West zone	7,399	20.05	10,827	19.65	18,226	17.48
Central zone	2,827	6.21	4,461	6.15	7,288	6.99
North-east zone	4,162	1.82	7,328	2.21	11,490	11.02
Union Territory	2,098	1.77	3,678	2.62	5,776	5.54
**Free Advice**						
Yes	17,085	38.19	31,229	45.84	48,314	46.35
No	23,371	61.81	32,552	54.16	55,923	53.65
**Marital Status**						
Unmarried	15,830	37.99	25,497	38.21	41,327	39.65
Married	24,626	62.01	38,288	61.79	62,914	60.35

Source: Based on the authors’ computation from NSSO 71st and 75th round unit level data. The 'N' represents the unweighted sample size of each category of the independent variable. '%' is the weighted percentage of each category of the independent variable for the cross-sectional data sets and the unweighted percentage for the pooled data set.

Various socio-economic and demographic variables are used in the study to see the adjusted impact of illness on hardship financing, all of which are categorical. The individual characteristics included in the analysis are the age group to which the person with illness belongs, sex, education status, type of employment, whether the person is suffering from any chronic diseases, type of medical institution approached during ailment, whether the person has received any fully or partially free medical advice, the marital, and insurance status of the person. We have not included an individual's insurance status as a control variable for outpatient care in India since outpatient cases often have limited insurance coverage. Various household characteristics are also included in the analysis. They are the household’s income quintile, living condition index, type of residence, religion, social group and state regions. The social group to which the household belongs is categorised as scheduled caste (SC), scheduled tribe (ST), other backward castes (OBC), and others. The living condition index is calculated using principle component analysis of information on the type of energy, drainage, latrine used, drinking water, and arrangement of disposal of garbage [5, 53]. The individual and household-level control variables used in the study can be summarised as given in the Additional file 1: Appendix Table 3.

**Table 3:** Logistic regression results of outpatient cases

**Outpatient**	**Model 1** **2014**		**Model 2** **2018**		**Model 3** **(Pooled)**	
**VARIABLES**	**Odds ratio**	**95% CI**	**Odds ratio**	**95% CI**	**Odds** **ratio**	**95% CI**
**Ailment**						
Infection®						
Cancer	6.11***	(2.95 - 12.64)	3.07***	(2.14 - 4.40)	3.53***	(2.57 - 4.83)
Blood Disease	1.70	(0.89 - 3.27)	1.34	(0.85 - 2.12)	1.45**	(1.00 - 2.11)
Endocrine	0.79	(0.58 - 1.06)	0.81**	(0.66 - 1.00)	0.81**	(0.68 - 0.96)
Psychiatric	1.53***	(1.11 - 2.10)	1.32**	(1.03 - 1.68)	1.40***	(1.15 - 1.70)
Genito-urinary	1.49	(0.91 - 2.44)	1.85***	(1.32 - 2.58)	1.75***	(1.33 - 2.30)
Eye	0.86	(0.45 - 1.64)	1.06	(0.66 - 1.70)	0.99	(0.68 - 1.44)
Ear	0.35	(0.09 - 1.45)	0.60	(0.24 - 1.50)	0.52*	(0.24 - 1.12)
Cardio-vascular	0.84	(0.63 - 1.11)	0.90	(0.74 - 1.10)	0.88	(0.75 - 1.04)
Respiratory	0.87	(0.68 - 1.12)	0.93	(0.76 - 1.14)	0.91	(0.78 - 1.06)
Gastro-intestinal	1.52***	(1.13 - 2.05)	1.22	(0.94 - 1.59)	1.36***	(1.12 - 1.66)
Skin	1.03	(0.64 - 1.67)	1.02	(0.70 - 1.50)	1.01	(0.75 - 1.36)
Musculo-Skeletal	1.03	(0.77 - 1.37)	1.08	(0.86 - 1.35)	1.07	(0.90 - 1.27)
Obstetric	3.81***	(1.89 - 7.68)	1.65	(0.82 - 3.30)	2.37***	(1.46 - 3.86)
Injuries	3.34***	(2.24 - 4.99)	2.33***	(1.71 - 3.17)	2.59***	(2.03 - 3.30)
Others	1.41	(0.91 - 2.19)	1.75***	(1.22 - 2.49)	1.62***	(1.23 - 2.13)
**Gender**						
Male®						
Female	0.98	(0.85 - 1.13)	0.90*	(0.82 - 1.00)	0.93*	(0.85 - 1.00)
**Education Status**						
Graduation and above ®						
Illiterate	2.29***	(1.50 - 3.49)	1.61***	(1.27 - 2.04)	1.75***	(1.43 - 2.15)
Less than primary education	2.40***	(1.56 - 3.71)	1.52***	(1.18 - 1.96)	1.72***	(1.38 - 2.13)
Completed primary education	2.25***	(1.46 - 3.46)	1.46***	(1.13 - 1.87)	1.62***	(1.31 - 2.01)
Middle school completed	1.95***	(1.26 - 3.01)	1.43***	(1.11 - 1.84)	1.52***	(1.23 - 1.89)
Completed secondary or higher secondary	1.27	(0.81 - 1.97)	1.16	(0.91 - 1.48)	1.18	(0.96 - 1.46)
**Type of Employment**						
Household without income®						
Casual labour	0.31***	(0.26 - 0.37)	0.25***	(0.22 - 0.29)	0.29***	(0.26 - 0.32)
Self-employed	0.39***	(0.31 - 0.48)	0.27***	(0.23 - 0.31)	0.32***	(0.28 - 0.36)
Regular wage	0.74***	(0.61 - 0.90)	0.29***	(0.25 - 0.33)	0.41***	(0.37 - 0.47)
**Income Quintile**						
Rich®						
Poorest	2.17***	(1.75 - 2.69)	1.54***	(1.31 - 1.81)	1.74***	(1.53 - 1.98)
Poor	1.26**	(1.02 - 1.56)	1.35***	(1.14 - 1.60)	1.30***	(1.14 - 1.48)
Middle	1.07	(0.84 - 1.36)	1.19**	(1.03 - 1.39)	1.17**	(1.03 - 1.33)
Richest	0.86	(0.69 - 1.05)	0.84**	(0.73 - 0.98)	0.84***	(0.74 - 0.95)
**Living condition Index**						
Low®						
High	0.83**	(0.70 - 0.99)	0.94	(0.84 - 1.06)	0.89**	(0.81 - 0.98)
**Chronic Diseases**						
Suffered®						
Did not suffer	0.92	(0.76 - 1.12)	1.01	(0.86 - 1.17)	0.97	(0.86 - 1.09)
**Location**						
Rural®						
Urban	0.99	(0.85 - 1.17)	0.95	(0.84 - 1.06)	1.02	(0.93 - 1.11)
**Religion**						
Others ®						
Hinduism	1.15	(0.72 - 1.82)	1.74***	(1.22 - 2.49)	1.48***	(1.12 - 1.96)
Islam	1.28	(0.79 - 2.09)	1.96***	(1.35 - 2.86)	1.67***	(1.24 - 2.24)
Christianity	1.68*	(0.99 - 2.87)	1.62**	(1.08 - 2.44)	1.61***	(1.17 - 2.22)
**Social Group**						
SC/ST ®						
OBC	1.23**	(1.03 - 1.47)	1.00	(0.87 - 1.13)	1.09	(0.98 - 1.21)
Others	1.02	(0.83 - 1.25)	0.96	(0.84 - 1.11)	1.00	(0.89 - 1.12)
**Type of Medical Institution**						
Public®						
Private	1.56***	(1.24 - 1.96)	0.72***	(0.62 - 0.83)	0.92	(0.82 - 1.04)
**Age Group**						
Children®						
Working age	1.46***	(1.13 - 1.89)	1.35***	(1.11 - 1.63)	1.39***	(1.20 - 1.62)
Elderly	1.73***	(1.33 - 2.24)	1.30***	(1.07 - 1.59)	1.45***	(1.24 - 1.70)
**State Regions**						
South zone ®						
North zone	0.62***	(0.50 - 0.78)	0.72***	(0.62 - 0.84)	0.70***	(0.62 - 0.79)
East zone	0.94	(0.78 - 1.14)	0.75***	(0.65 - 0.86)	0.81***	(0.72 - 0.91)
West zone	0.63***	(0.50 - 0.80)	0.53***	(0.44 - 0.63)	0.56***	(0.49 - 0.65)
Central zone	0.67**	(0.47 - 0.96)	0.81*	(0.64 - 1.02)	0.77***	(0.64 - 0.94)
North-east zone	0.22***	(0.10 - 0.50)	0.46***	(0.32 - 0.65)	0.39***	(0.28 - 0.53)
Union Territories	0.66**	(0.44 - 0.99)	0.31***	(0.23 - 0.42)	0.40***	(0.32 - 0.51)
**Free Advice**						
Yes®						
No	0.89	(0.71 - 1.12)	0.51***	(0.44 - 0.59)	0.60***	(0.53 - 0.68)
**Marital Status**						
Unmarried ®						
Married	0.90	(0.76 - 1.06)	0.87**	(0.77 - 0.97)	0.88***	(0.80 - 0.96)
**Year**						
2014®						
2018					1.51***	(1.38 - 1.64)
Constant	0.02***	(0.01 - 0.04)	0.13***	(0.08 - 0.21)	0.05***	(0.03 - 0.08)
Observations	26,757		38,188		64,945	

Source: Based on the authors’ calculation from NSSO 71^st^ and 75th rounds. Note: Confidence interval in parenthesis. *** represents *p*<0.01, ** represents *p*<0.05, * represents *p*<0.1

### Statistical methods

**Fig. 2:**
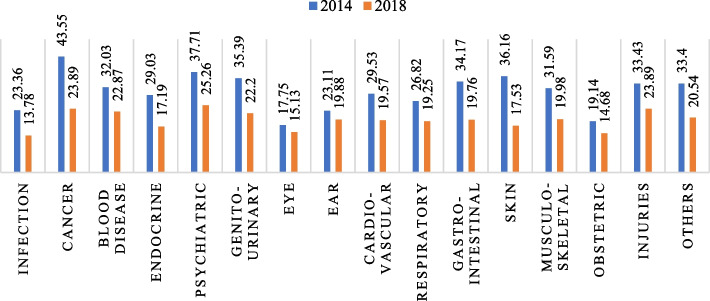
Disease-specific hardship financing for inpatient cases in 2014 and 2018. Source: Based on the authors’ calculation using NSSO 71st and 75th rounds

**Fig. 3:**
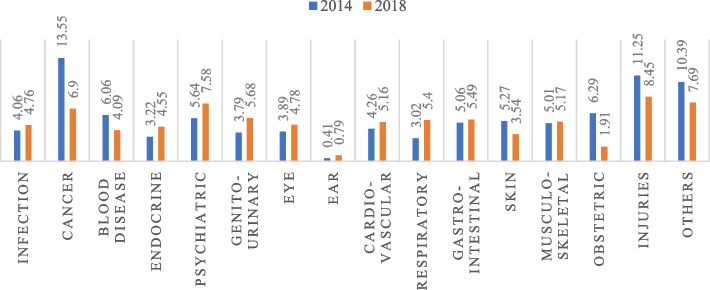
Disease-specific hardship financing for outpatient cases in 2014 and 2018. Source: Based on the authors’ calculation using NSSO 71st and 75th rounds

**Fig. 4:**
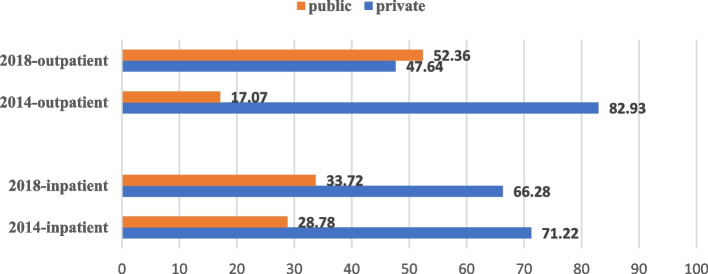
Percentage difference in hardship financing in public and private care sectors. Source: Based on the authors’ calculation using NSSO 71st and 75th rounds

Descriptive statistics are reported, and multivariate analyses are carried out to explain the objectives. Firstly, descriptive statistics are used to get the preliminary results. Descriptive statistics are reported to understand the distribution of the study population. Graphical representation of the percentage distribution of hardship financing across disease groups (figure 2 and figure 3) and private and public health facilities (figure 4) are provided. The sample size and percentage distribution of each category of the independent variables are reported (Tables 1 and 2).

Secondly, logistic regression is estimated, and odds ratios are reported to understand the adjusted impact of illness on hardship financing. Regression analysis is done separately for the inpatient and outpatient cases who received medical attention at both periods. The regression models, namely model 1, model 2 for outpatients (in Table 3), model 4, and model 5 for inpatients (in Table 4), represent the cross-sectional regression models for 2014 and 2018, respectively.

**Table 4:** Logistic regression of inpatient cases

**Inpatient**	**Model 4** **2014**		**Model 5** **2018**		**Model 6** **(Pooled)**	
**VARIABLES**	**Odds ratio**	**95% CI**	**Odds ratio**	**95% CI**	**Odds ratio**	**95% CI**
**Ailment**						
Infection®						
Cancer	2.41***	(2.03 - 2.86)	2.54***	(2.21 - 2.93)	2.49***	(2.23 - 2.78)
Blood Disease	1.49***	(1.25 - 1.77)	1.61***	(1.37 - 1.89)	1.56***	(1.39 - 1.76)
Endocrine	1.26***	(1.08 - 1.48)	1.08	(0.93 - 1.26)	1.17***	(1.05 - 1.30)
Psychiatric	1.70***	(1.53 - 1.90)	1.68***	(1.52 - 1.86)	1.69***	(1.57 - 1.82)
Genito-urinary	1.64***	(1.47 - 1.82)	1.65***	(1.50 - 1.82)	1.65***	(1.54 - 1.77)
Eye	0.78***	(0.68 - 0.91)	1.00	(0.88 - 1.15)	0.89**	(0.80 - 0.98)
Ear	1.11	(0.75 - 1.64)	1.31*	(0.96 - 1.80)	1.22	(0.95 - 1.56)
Cardio-vascular	1.43***	(1.28 - 1.58)	1.40***	(1.28 - 1.53)	1.41***	(1.32 - 1.51)
Respiratory	1.16**	(1.03 - 1.31)	1.28***	(1.13 - 1.44)	1.22***	(1.11 - 1.33)
Gastro-intestinal	1.41***	(1.29 - 1.55)	1.54***	(1.42 - 1.67)	1.48***	(1.40 - 1.58)
Skin	1.50***	(1.18 - 1.90)	1.21	(0.95 - 1.54)	1.36***	(1.15 - 1.61)
Musculo-Skeletal	1.32***	(1.16 - 1.49)	1.50***	(1.34 - 1.67)	1.42***	(1.31 - 1.54)
Obstetric	1.14*	(0.99 - 1.30)	1.41***	(1.22 - 1.65)	1.24***	(1.13 - 1.37)
Injuries	1.78***	(1.63 - 1.95)	1.72***	(1.59 - 1.85)	1.73***	(1.63 - 1.83)
Others	1.60***	(1.38 - 1.86)	1.71***	(1.48 - 1.97)	1.66***	(1.50 - 1.84)
**Gender**						
Male®						
Female	0.79***	(0.75 - 0.84)	0.83***	(0.79 - 0.87)	0.81***	(0.78 - 0.84)
**Education Status**						
Illiterate®						
Less than primary education	0.93*	(0.86 - 1.01)	0.87***	(0.81 - 0.94)	0.90***	(0.85 - 0.95)
Completed primary education	0.86***	(0.79 - 0.93)	0.87***	(0.81 - 0.94)	0.86***	(0.82 - 0.91)
Middle school completed	0.86***	(0.80 - 0.94)	0.79***	(0.73 - 0.85)	0.82***	(0.78 - 0.87)
Completed secondary or higher secondary	0.68***	(0.63 - 0.74)	0.70***	(0.65 - 0.75)	0.69***	(0.65 - 0.73)
Graduation and above	0.45***	(0.40 - 0.52)	0.52***	(0.47 - 0.58)	0.50***	(0.46 - 0.54)
**Type of Employment**						
Casual labour®						
Self-employed	0.67***	(0.63 - 0.72)	0.68***	(0.64 - 0.72)	0.69***	(0.66 - 0.72)
Regular wage	0.68***	(0.62 - 0.74)	0.67***	(0.63 - 0.72)	0.68***	(0.64 - 0.72)
Household without income	0.89**	(0.82 - 0.98)	1.35***	(1.23 - 1.47)	1.10***	(1.03 - 1.16)
**Income Quintile**						
Middle®						
Poorest	1.32***	(1.20 - 1.44)	1.17***	(1.09 - 1.25)	1.23***	(1.17 - 1.30)
Poor	1.11**	(1.02 - 1.21)	1.10***	(1.02 - 1.18)	1.10***	(1.04 - 1.16)
Rich	0.94	(0.86 - 1.02)	0.91***	(0.85 - 0.97)	0.92***	(0.87 - 0.97)
Richest	0.70***	(0.65 - 0.77)	0.73***	(0.68 - 0.78)	0.71***	(0.68 - 0.75)
**Living condition Index**						
Low®						
High	0.63***	(0.59 - 0.67)	0.79***	(0.74 - 0.83)	0.70***	(0.67 - 0.73)
**Insurance**						
Insured®						
Uninsured	0.93**	(0.87 - 0.99)	0.69***	(0.65 - 0.72)	0.78***	(0.75 - 0.81)
**Chronic Diseases**						
Suffered®						
Did not suffer	0.74***	(0.70 - 0.79)	0.71***	(0.67 - 0.76)	0.73***	(0.69 - 0.76)
**Location**						
Urban®						
Rural	1.04	(0.98 - 1.10)	1.01	(0.95 - 1.07)	1.00	(0.96 - 1.04)
**Religion**						
Islam ®						
Hinduism	0.86***	(0.80 - 0.92)	1.00	(0.94 - 1.08)	0.94***	(0.89 - 0.98)
Christianity	0.83**	(0.72 - 0.97)	0.99	(0.87 - 1.13)	0.92*	(0.83 - 1.01)
Others	0.82**	(0.70 - 0.98)	0.94	(0.81 - 1.09)	0.90*	(0.81 - 1.01)
**Social Group**						
SC/ST ®						
OBC	0.89***	(0.84 - 0.95)	0.94**	(0.88 - 0.99)	0.92***	(0.88 - 0.96)
Others	0.76***	(0.71 - 0.82)	0.85***	(0.80 - 0.91)	0.81***	(0.78 - 0.86)
**Type of Medical Institution**						
Public®						
Private	1.70***	(1.56 - 1.84)	1.53***	(1.42 - 1.66)	1.59***	(1.51 - 1.69)
**Age Group**						
Working age®						
Children	0.83***	(0.76 - 0.91)	0.77***	(0.71 - 0.83)	0.80***	(0.75 - 0.85)
Elderly	0.74***	(0.69 - 0.80)	0.80***	(0.75 - 0.86)	0.78***	(0.75 - 0.82)
**State Regions**						
South zone ®						
North zone	0.38***	(0.35 - 0.41)	0.59***	(0.55 - 0.64)	0.49***	(0.46 - 0.52)
East zone	0.61***	(0.56 - 0.65)	0.64***	(0.60 - 0.69)	0.62***	(0.59 - 0.66)
West zone	0.35***	(0.33 - 0.38)	0.49***	(0.46 - 0.53)	0.43***	(0.41 - 0.45)
Central zone	0.45***	(0.40 - 0.50)	0.58***	(0.53 - 0.64)	0.53***	(0.49 - 0.56)
North-east zone	0.14***	(0.12 - 0.16)	0.26***	(0.23 - 0.29)	0.20***	(0.18 - 0.22)
Union Territory	0.25***	(0.21 - 0.29)	0.30***	(0.26 - 0.35)	0.28***	(0.25 - 0.31)
**Free Advice**						
Yes®						
No	1.39***	(1.29 - 1.51)	1.13***	(1.05 - 1.22)	1.24***	(1.18 - 1.31)
**Marital Status**						
Unmarried ®						
Married	0.93**	(0.87 - 0.99)	0.90***	(0.85 - 0.95)	0.91***	(0.87 - 0.95)
**Year**						
2014®						
2018					0.58***	(0.56 - 0.60)
Constant	1.23**	(1.04 - 1.45)	0.68***	(0.59 - 0.78)	1.21***	(1.08 - 1.35)
Observations	40,451		63,780		104,231	

Source: Based on the authors’ calculation from NSSO 71^st^ and 75th rounds. Note: Confidence interval in parenthesis. *** represents *p*<0.01, ** represents *p*<0.05, * represents *p*<0.1

Thirdly pooled logistic regression is conducted using the pooled cross-section data from 2014 to 2018 to check the robustness of the cross-sectional analyses (model 3 and model 6 for outpatients and inpatients, respectively). A year variable is introduced in this regression model to distinguish between the periods. The pooled logistic regression model can be summarised as in equation (1):

1 $$Ln(\frac{{p}_{i}}{1-{p}_{i}})={\beta }_{0}+{\upbeta }_{1}{X}_{ij}+{\beta }_{2}{Y}_{ij}+{\beta }_{3}{Z}_{ij}+{\beta }_{4}{T}_{i}+{\epsilon }_{0}$$

where ${p}_{i}=$ probability of success/ hardship financing (coded as1),

$1-{p}_{i}=$ probability of failure/ no hardship financing (coded as 0),

${X}_{ij}=$ classification of Illnesses,

${Y}_{ij}=$ vector of individual characteristics,

${Z}_{ij}=$ vector of household characteristics,

$${T}_{i}=$$ year dummy,

$${\epsilon }_{0}=$$ error term.

Fourthly the average marginal effects are calculated from the pooled models to understand the direction and magnitude of change in hardship financing from 2014 to 2018. Additional to the robustness of the model, we also tested for multicollinearity in the cross-sectional models and found that the mean-variance inflation factor is less than 3. Therefore our models do not suffer from severe multicollinearity issues. We have not used weights in the regression analysis as NSSO is large-scale data representing the nation. The statistical analyses in the study are performed using the STATA software version 12.0.

## Results

### Descriptive statistics

From 2014 to 2018, the rate of inpatient cases resorting to informal distress financing decreased from 29% to 18.47%, whereas the percentage of outpatients increased from 4.36% to 5.08%. Figures 2 and 3 display the percentage distribution of hardship financing among the disease groups for inpatients and outpatients, respectively, comparing the two periods. Around 44% of inpatient and 14% of outpatient cancer cases faced the highest percentage of hardship financing in India in 2014. From 2014 to 2018, the economic hardship across all the disease groups decreased for inpatients. Whereas for outpatients, the percentage change across diseases is not unidirectional. For some disease groups, namely infectious diseases, cancer, blood diseases, skin diseases, obstetrics, injuries and other illnesses, the percentage of the disease-specific cases suffering from hardship financing has decreased, whereas, for the rest of the disease groups, it has increased.

Figure 4 illustrates how the percentage of hardship financing varied across the people who accessed care in the private versus public sectors in the two periods. In 2014, the percentage of cases resorting to hardship financing in the public sector was lower than that of the private sector for inpatients and outpatients. In 2018 for inpatients, the hardship financing in the public sector (33.72%) was lower than in the private sector (66.28%). Whereas for outpatients in 2018, the percentage of cases resorting to hardship financing is higher in the public sector (52.36%) than in the private sector (47.64%).

Tables 1 and 2 give the socio-economic, demographic characteristics, and disease classification of outpatient and inpatient cases, respectively. In Table 1, there are 26,763 cases in the 71^st^ round and 38,217 patients in the 75^th^ round and the pooled data of both rounds have a sample size of 64,980. In Table 1, compared to infectious diseases, the reported number of cases for non-communicable illnesses like cancer was significantly less. After infectious diseases (27% in the 71^st^ and 31% in the 75^th^ round), the second biggest disease share of outpatients was cardiovascular diseases (around 15% in the 71^st^ and 18% in the 75^th^ round). It is followed by endocrine diseases (about 14% in the 71^st^ round and 16% in the 75^th^ round). More than half the cases of outpatients were women, around 55% in both rounds. The patients with no literacy or formal schooling were the highest (about 38% in 71^st^ and 35% in the 75^th^ round), and people with a graduation degree or above were the lowest (around 6% in 71^st^ and 8% in the 75^th^ round). There was a large proportion of self-employed adults, roughly 45%, in both rounds. Nearly 17% and 20% of people belonged to the poorest 20% quintile in rounds 71 and 75, respectively. Approximately 55% of outpatient cases suffered from chronic diseases in both periods. Roughly 78% of patients practised Hinduism, about 22% belonged to a Scheduled caste or tribe, and more than 60% belonged to the rural sector. About 70% of the cases approached the private sector for outpatient care, and around half of the patients in the 71^st^ round had a low living condition index. About 53% of the working-age adults were in the study, and 57% were married in both rounds. Among the state regions, the south zone had the highest percentage of cases of illness (around 40% in the 71^st^ and 31% in the 75^th^ round), and approximately three out of four cases had not received free medical advice.

In Table 2 representing inpatient cases, about one out of four cases in the 71^st^ round suffered from infectious diseases. After infectious diseases, the second most significant percentage of the study population had injuries (around 11%). It is followed by gastrointestinal diseases (11% in the 71^st^ round and 10% in the 75^th^ round). Approximately half the study population were women. The most significant number of cases were illiterate (37 % in the 71^st^ round and 32 % in the 75^th^ round), and the least were with graduate-level education or more (around 6%). Self-employment was the most usual type of employment among the ill population at approximately 48%. About 15% of cases belonged to people in the lowest 20% quintile, and about 55% of individuals had a low living condition index in the 71^st^ round. About 75% and 80% of cases in the 71^st^ and 75^th^ rounds had not suffered from chronic conditions, and around 60% of the ill people approached the private sector for inpatient-level care. Uninsured individuals constituted three out of four inpatient cases. In the 71^st^ round, only 35% of patients belonged to rural areas, whereas in 75^th^, 65% belonged there. Around 80% of inpatients follow Hinduism, and 1 out of 4 belonged to SC/ST social group. Approximately 62% belonged to the working age group, and the maximum number of cases were from the south zone. About 62% of patients are married people. Additionally, 62% and 54% of individual inpatient cases had not received free medical advice in 71^st^ and 75^th^ rounds, respectively.

### Multivariate analysis

The regression analysis has more than one outcome variable and predictor variables, making it the multivariate multiple regression model [54]. Tables 3 and 4 are the logistic regression models which give the association between the explanatory variables and hardship financing for individual cases of illness.

### Outpatient cases

The analysis shows that cancer cases compared to infectious diseases had higher odds of facing hardship financing (Odds ratio: 6.11 in 2014 and 3.07 in 2018) at a 1 per cent significance level. Women were less likely to incur hardship financing than men, at a 10 per cent significance level in 2018. In our results, the increase in education level made it less likely to face hardship financing. Compared to individuals with an education level of graduation or more, illiterate people were 2.29 times more likely in 2014 and 1.61 times more likely in 2018 to face hardship financing. In 2018, Hindus, Christians and Muslims had a significantly higher likelihood than other religions. Muslims were most likely to suffer from distress financing. And compared to SC/ST, OBC was 1.23 times more likely to face hardship financing in 2014. The working-age population and the elderly had a significantly higher likelihood than children to face hardship financing. Married people were significantly less likely to resort to hardship financing than unmarried. Compared to the southern zone, every other region in the country was considerably less likely to suffer from hardship financing.

Compared to households without income, regular-wage households were around 0.3 and 0.7 times less likely to suffer from hardship financing in 2014 and 2018, respectively. The household income quintiles and living condition index show a similar pattern here. Households from a wealthier quintile and better living condition index were less likely to suffer from hardship financing. The poorest quintile was 2.17 times more likely to suffer from hardship financing in 2014.

The likelihood of incurring hardship financing was 1.56 times more likely in 2014 and 0.3 times less likely in 2018 among people utilising private hospitals compared to public hospitals. And finally, those who did not get free medical advice were less likely to face hardship financing.

### Inpatient cases

Compared to infectious diseases, the odds of facing hardship financing for cancer cases were 2.41 and 2.54 times more likely in 2014 and 2018, respectively. Individuals without chronic diseases were 0.3 times less likely to face hardship financing than those with chronic illnesses.

Women were 0.2 times less likely to face hardship financing compared to men. The higher the education level, the lower the likelihood of hardship financing. People in rural areas had a higher likelihood of hardship financing than in urban areas. Unmarried people were more likely to suffer than married people. Compared to Islam, every other religion was less likely to face distress financing in 2014. SC/ST population was more likely to suffer than other social groups, and the children and the elderly were less likely to suffer compared to the working-age group from hardship financing. The south zone had the highest likelihood of distress financing compared to other regions.

Like outpatient cases, with the increase in income, and better living condition index, the likelihood of hardship financing decreased. Households with regular wages were 0.3 times less likely to face hardship financing than casual labourers.

People who accessed private hospitals were more likely to suffer from hardship financing than those who used public hospitals. Uninsured individuals were less likely to have encountered hardship financing than insured cases. Unlike outpatient cases, people who did not receive free advice were 1.39 and 1.13 times more likely to face hardship financing than those who received it in 2014 and 2018, respectively.

### Robustness of the model

Robustness analysis is done using pooled logistic regression [55]. The pooled cross-sectional analysis combines the information on two time periods separately for outpatients and inpatients, model 3 and model 6, from Tables 1 and 2, respectively. The results from these models suggest that most of the variables used in the analysis are relevant, and the results offered are similar to the cross-sectional models.

In the outpatient model, there was some ambiguity between the two time periods for the independent variables: treatment institution, ailment in the eye, chronic diseases and location of stay. Among treatment institutions, the likelihood of hardship financing among private institutions was higher than public institutions in 2014; however, the results show it was less likely to cause hardship financing in 2018. Compared to infectious diseases, the ailment of the eye was less likely to cause hardship financing in 2014. In contrast, in 2018, the likelihood of eye diseases causing hardship financing was the same as infectious diseases. Among cases of chronic diseases, in 2014, those who did not have a chronic illness were less likely to cause hardship financing than those who suffered, whereas, in 2018, the cases that did not suffer were more likely to cause it. The cases from rural areas in both 2014 and 2018 were slightly less likely to cause hardship financing than urban cases; however, the pooled regression shows more likelihood of hardship financing among rural cases. However, the odds ratios reported for chronic diseases and location of residence are insignificant. In the inpatient model, every variable is robust except for a predictor category household without income.

### Marginal effects

Table 5 is a marginal effects model that gives the average marginal impact from 2014 to 2018. In the year 2018, the probability of hardship financing by inpatient cases decreased by 7.5%. In contrast, for outpatient cases, in the year 2018 the probability of hardship financing increased by 1.3%. Both results are highly statistically significant.

**Table 5:** Marginal effects model

Year	${\varvec{d}}{\varvec{y}}/{\varvec{d}}{\varvec{x}}$	CI
2018 (outpatients)	0.0126***	0.01 - 0.02
2018 (inpatients)	-.0747***	-0.80 - -0.70

Source: Based on the authors’ calculation from NSSO 71^st^ and 75th rounds. Note: Confidence interval in parenthesis. *** represents *p*<0.01, ** represents *p*<0.05, * represents *p*<0.1

## Discussion

The healthcare costs and the loss of health from sickness lead to poverty and often a medical poverty trap [36]. The rising dual burden of diseases in a predominantly developing country warrants looking into the disease-specific impact of illness and the informal financial mechanisms people adopt to cope with them over time. With the epidemiological transition, there has been a massive increase in cancer patients in India, and in 2018, an additional million new patients were identified [56]. Our study results show that of all the diseases, cancer was most likely to make people resort to hardship financing in India. We can find similar results in studies which point out that the highest hospitalisation expenditure is for cancer [32, 35, 57]. Since 2010, the government has launched programs at the district level for the prevention and control of Cancer, Diabetes, Cardiovascular Diseases and Stroke (NPCDCS) [58]. However, the failure of proper implementation and monitoring has left the program unable to improve its coverage [5].

When men and women are exposed to the same illness, the biological risk of contracting them varies across the sexes [59]. However, these biological differences are insufficient to explain the striking imbalance in the gendered response to illnesses[60, 61]. The statistics on higher life expectancy at birth for women mask the reality of high mortality rates among them [62–65]. The intra-household financial response to illnesses also varies across gender [66]. In our study, the percentage of ill women was approximately 50% or higher; however, illness episodes among men were more prone to hardship financing than women.

According to Wagstaff, in Vietnam, health shocks, especially the death of a working-age member had a severe negative impact on household income [67]. Kochar [22] points out that labour provided by a non-disabled male household member is a means of insurance against crop shock in the household. The present study agrees with this literature on the importance of the working-age population as an essential household income source. Approximately 53% of outpatient and 62% of inpatient cases belonged to the working-age population. Our results show that a health shock faced by the working-age population was more likely than among children or the elderly to cause hardship financing in India. Gertler & Gruber [25] point out that illness's impact on consumption is lesser when the head of the household is more educated. The results from our study show that with higher education, it is less likely for the illness to lead to distress financing compared to illiterate people.

In our results, households without an income source were most likely to face hardship financing in India. Also, people from the poorest quintile were more susceptible to hardship financing than rich households. Past works show similar findings where poor households were more vulnerable than rich households [5, 31, 37, 49, 68]. Our study suggests that cases in rural areas were more likely to cause distress financing. Verma et al. [5] find higher OOPE and distress financing among the rural population. There is evidence that state-sponsored health insurance schemes did not reduce the welfare loss arising from health shocks and increased the risk of catastrophic health spending among households with health insurance [69–71]. Our study results agree with these results from China and USA. In India, the coverage offered by insurance schemes was minimal. In our study for outpatient and inpatient cases, individuals with health insurance were more likely to face hardship financing when compared to uninsured individuals. According to Gertler and Gruber [25], the impact of illness on consumption depends on the intensity of illness shock. The significant and severe health shock often hinders households' ability to insure consumption. In India, chronic diseases aren't covered by many existing schemes and can result in catastrophic health expenditures [32]. Our results suggest that people who suffered from chronic diseases were more likely to incur hardship financing than those who did not.

In our marginal effects model, from 2014 to 2018, there had been a fall in the percentage of people resorting to hardship financing for inpatient cases. However, in the case of outpatients, the model shows that hardship financing has increased marginally. The National Health Policy 2017 recommends spending about 2/3rd of the health budget on primary healthcare [72]. The government health expenditure on inpatient and outpatient increased by almost 4% between 2014 and 2018 [1, 73]. In 2018, the share of government and compulsory contributory health care financing schemes on inpatient curative care was 11.6% of current health expenditure, and outpatient curative care was 10.32% of current health expenditure [1].

Our study results show that among outpatients in 2018, hardship financing was lower for people who went to private sector providers compared to the public sector. The literature indicates that the expenditure on drugs and pharmaceuticals must have contributed to the higher hardship financing in the public outpatient sector. The out-of-pocket payment for hospitalisation is not the primary cause of poverty in India. Expenditure on drugs is the primary cause, and 80 per cent of this expenditure falls on outpatient care and only about 20 per cent on inpatient care [15]. For outpatients, medicines contributed to a mean of 60.3% of OOPE [74]. In Odisha, out of all outpatient visits, 86% of them resorted to private sector pharmacies [75, 76]. Even when the public sector is supposed to provide medicine for free, about 70% of people who use public sector hospitals and primary health facilities have bought their medicines from private pharmacies [76].

We put forward some limitations of the study. Firstly, we have done a separate analysis for inpatient and outpatient cases. These results are not comparable because of the difference in their recall periods. For inpatients, the recall period last 365 days, whereas for outpatients, the recall period is only the previous 15 days. The recall bias could be higher for inpatients as the recall period is very long. Secondly, our data source only provides information on whether hardship financing was practised for a particular ailment. We do not have the amount of money that was raised by borrowing or selling. Thus, we are not able to quantify the extent of hardship financing. Thirdly, hardship financing is self-reported data and can be subject to bias. Evidence suggests that people are often reluctant to admit to mishandling their economic resources or confessing that they face financial problems [77, 78]. Fourthly, we have excluded cases reporting child delivery from our study. Our rationale for doing so is that childbirth is not an unanticipated health shock. Fifthly, reported cases of non-communicable diseases like cancer are very few for outpatients. When diseases are self-reported, there is a problem of under or over-estimation [25, 79, 80]. Evidence shows that outpatient care for NCDs is 50% more expensive than for communicable diseases [81]. Thus, there is a possibility of underreporting the cases due to foregone care because of the cost. Even the socio-economic background impacts the reporting of illness. Often, educated, wealthier, and socially advanced people are more likely to report symptoms of illness in the last month as they are more sensitive to the limitations of health loss [25, 82].

### Conclusion and scope for future research

According to the evidence from the current study, cancer cases continue to result in the maximum hardship financing in India over the years. Conflicting results are drawn from the study on the direction of change in hardship financing in India between 2014 and 2018. While hardship financing has decreased for inpatients, it has increased for outpatient cases. Additionally, in 2018 among outpatients, the hardship financing among cases that relied on public health facilities is higher than those who approached the private health sector.

Thus, the government's attempts to counteract the financial risk to health seem insufficient, and people rely on informal coping mechanisms when they experience a health shock. Our empirical results are similar to the existing studies on hardship financing related to the evidence of high OOPE on outpatient medicine expenditure and urge policymakers to make outpatient care more financially accessible to the public.

The current evidence also shows that in Indian households, hardship financing for men's health care needs receives priority over women's. Future research can be focused on identifying inequalities among households and link with hardship financing. With available data, it would be essential to explain the intra-household differences in hardship financing related to various types of illness. Therefore it is pertinent to conduct further research to understand how the household’s strategies to borrow or sell vary across gender and other socioeconomic parameters.

## Supplementary Information

**Additional file 1.**

## Data Availability

The data sets generated and/or analysed in the current study are available in the public domain. They can be downloaded on request from the National Data Archive of the Ministry of Statistics and Program Implementation, Government of India. The NSS 71^st^ round data on Social Consumption: Health can be downloaded using the link: http://microdata.gov.in/nada43/index.php/catalog/135/get_microdata. And the NSS 75^th^ round data on Household Social Consumption: Health can be downloaded from: http://microdata.gov.in/nada43/index.php/catalog/152.
