# Supplementary material for: Illnesses and hardship financing in India: an evaluation of inpatient and outpatient cases, 2014-18
Source: BMC Public Health. 2023 Jan 30;23:204. doi: 10.1186/s12889-023-15062-7 (PMC9887799; doi:10.1186/s12889-023-15062-7)
Supplement: Supplementary file 1 — Additional file 1. [file 12889_2023_15062_MOESM1_ESM.docx]

**Appendix Table 1: Expanded Range of Services under HWC**

| RMNCH+A | Provision of care during the gestation period and delivery |
| --- | --- |
|  | Services for neonatal and infant needs |
|  | Services for children and adolescent youth. |
|  | Contraception, family planning programs |
| Communicable diseases | National health program |
|  | Gate keeper role by provision of out-patient care for those communicable diseases which are common and other minor ailments. |

+

| Non communicable diseases (NCDs) | Screening for prevention and treatment of chronic NCDs like TB |
| --- | --- |
|  | Basic Dental Care |
|  | Ophthalmology and ENT services |
|  | Geriatric care |
|  | Emergency care like during an accident or other such injuries. |
|  | Mental health |

Source: based on information provided at Ayushman Bharat Health and Wellness Centre website obtained from: https://ab-hwc.nhp.gov.in/

**Appendix Table 2: Disease Categorised as per NSSO 71^st^ and 75^th^ round report**

| **SI no.** | **Disease-groups** | **Main symptoms or diagnosis as reported in NSSO 75^th^ round (2018).** |
| --- | --- | --- |
| 1 | Infections | Fever with loss of consciousness or altered consciousness/ Malaria/ Fever from diphtheria or whooping cough/ all other fevers (includes typhoid, fever with rash/eruptive lesions and fever of unknown origin, all specific fevers without a confirmed diagnosis.) |
| 2 | Cancers | Cancers (known or suspected by a physician) and any growing painless lump in the body. |
| 3 | Blood diseases | Any case of anaemia/Bleeding disorders |
| 4 | Endocrine/Metabolic/  Nutritional | Diabetes/ Under-nutrition/ Goitre and other thyroid diseases/others (including obesity) |
| 5 | Psychiatric & Neurological | Mental retardation/Mental disorders/ Headache/ Seizures or known epilepsy/ Weakness in limb muscles and difficulty in movements/ Stroke or hemiplegia or sudden onset weakness or loss of speech in half of body/Others including memory loss, confusion. |
| 6 | Genito-urinary | Any difficulty or abnormality in urination/pain in the pelvic region or reproductive tract infection or pain in the male genital area/ Change or irregularity in the menstrual cycle or excessive bleeding or pain during menstruation and any other gynaecological and andrological disorders, including male or female infertility. |
| 7 | Eye | Discomfort or pain in the eye with redness or swellings or boil/Cataract/Glaucoma |
| 8 | Ear | Earache with discharge or bleeding from ear or infections/Decreased hearing or loss of hearing. |
| 9 | Cardio-vascular | Hypertension/Heart disease: Chest pain, breathlessness |
| 10 | Respiratory | Acute respiratory infections (cold, runny nose, sore throat with cough, allergic colds included)/Cough with sputum with or without fever and not diagnosed as TB/Bronchial asthma/recurrent episode of wheezing and breathlessness with or without cough over long periods or known asthma). |
| 11 | Gastro-intestinal | Diseases of mouth or teeth or gums/Pain in abdomen: gastric and peptic ulcers or acid reflux or acute abdomen lump or fluid in abdomen or scrotum/Gastrointestinal bleeding. |
| 12 | Skin | Skin infection (boil, abscess, itching) and other skin diseases. |
| 13 | Musculoskeletal | Joint or bone diseases or pain or swelling in any of the joints, swelling or pus from the bones/Back or body aches |
| 14 | Injuries | Accidental injury, road traffic accidents and falls/ Accidental drowning and submersion/Burns and corrosions/Poisoning/Intentional self-harm/Assault/Contact with venomous/harm causing animals and plants. |
| 15 | Obstetric | Pregnancy with complications before or during labour (abortion, ectopic pregnancy, hypertension, complications during delivery)/Illness in the newborn or sick newborn |
| 16 | Others | Symptoms are not fitting into any of the above categories/ Could not even state the main symptom. |

Source: Based on NSSO 71^st^ and 75^th^ rounds report on Social Consumption on Health

**Appendix Table 3: Description of Individual and Household level control variables**

| **SI no.** | **Variables** | **Description** |
| --- | --- | --- |
| **Individual Characteristics** | | |
| 1 | Age Group | child (0-14 years) |
|  |  | working age (15-59 years) |
|  |  | elderly(60 or above) |
| 2 | Sex | Male |
|  |  | Female |
| 3 | Education Status | Illiterate |
|  |  | Less than primary education |
|  |  | Completed primary education |
|  |  | Completed middle school |
|  |  | Completed secondary or higher education |
|  |  | Graduation and above |
| 4 | Type of Employment | Casual labour |
|  |  | Self-employed |
|  |  | Regular wage |
|  |  | Household without income |
| 5 | Suffered from Chronic Disease(s) | Suffered |
|  |  | Did not suffer |
| 6 | Medical Institution Used During Ailment | Public |
|  |  | Private |
| 7 | Free Medical Advice Status | Yes |
|  |  | No |
| 8 | Marital Status | Married |
|  |  | Unmarried |
| 9 | Insurance Status | Insured |
|  |  | Uninsured |
| **Household Level Characteristics** | | |
| 10 | Income Quintile | Poorest |
|  |  | Poor |
|  |  | Middle |
|  |  | Richer |
|  |  | Richest |
| 11 | Living condition index | Low |
|  |  | High |
| 12 | Type of Residence | Rural |
|  |  | Urban |
| 13 | Religion | Hinduism |
|  |  | Christianity |
|  |  | Muslim |
|  |  | Others |
| 14 | Social Group | SC/ST |
|  |  | OBC |
|  |  | Others |
| 15 | State categories | North zone |
|  |  | South zone |
|  |  | East zone |
|  |  | West zone |
|  |  | Central zone |
|  |  | North-east zone |
|  |  | Union territories |
| 16 | Year | 2014 |
|  |  | 2018 |
